# Unusual cerebral intraventricular hemorrhage and cardiomyopathy related to congenital cytomegalovirus from non-primary maternal infection: a case report

**DOI:** 10.1186/s13052-024-01637-6

**Published:** 2024-04-16

**Authors:** Victoria Malherbe, Stefanie Celen, Katherine Carkeek, Evelina Carapancea, Cinzia Auriti, Fiammetta Piersigilli

**Affiliations:** 1 Department of Pediatrics, Cliniques Universitaires St Luc, Brussels, Belgium; 2 Department of Neonatology, Cliniques Universitaires St Luc, Université Catholique de Louvain, Brussels, Belgium; 3 Department of Pediatric Neurology, Cliniques Universitaires St Luc, Institute of NeuroScience, Université Catholique de Louvain, Brussels, Belgium; 4 Saint Camillus International University of Health Sciences, Rome, Italy; 5 Department of Neonatology, Villa Margherita Private Clinic, Rome, Italy; 6 Neonatal intensive care unit, Department of Neonatology, Cliniques Universitaires St Luc, Avenue Hippocrate 10, 1200 Brussels, Belgium

**Keywords:** Congenital cytomegalovirus, Hypothermia, Status epilepticus, Universal screening, Case report

## Abstract

**Background:**

Congenital cytomegalovirus (cCMV) infection, resulting from non-primary maternal infection or reactivation during pregnancy, can cause serious fetal abnormalities, complications in the immediate neonatal period, and severe sequelae later in childhood. Maternal non-primary cytomegalovirus infection in pregnancy is transmitted to the fetus in 0.5-2% of cases (1).

**Case presentation:**

An African full term male newbornwas delivered by emergency caesarean section. Due to signs of asphyxia at birth and clinical moderate encephalopathy, he underwent therapeutic hypothermia. Continuous full video-electroencephalography monitoring showed no seizures during the first 72 h, however, soon after rewarming, he presented refractory status epilepticus due to an intracranial hemorrhage, related to severe thrombocytopenia. The patient also presented signs of sepsis (hypotension and signs of reduced perfusions). An echocardiography revealed severe cardiac failure with an ejection fraction of 33% and signs suggestive of cardiomyopathy. Research for CMV DNA Polymerase Chain Reaction (PCR) on urine, blood, cerebrospinal fluid, and nasopharyngeal secretions was positive.The mother had positive CMV IgG with negative IgM shortly before pregnancy. Serology for CMV was therefore not repeated during pregnancy, but CMV DNA performed on the Guthrie bloodspot taken at birth yielded a positive result, confirming the intrauterine transmission and congenital origin of the infection. The baby was discharged in good general condition and follow up showed a normal neurodevelopmental outcome at 9 months.

**Conclusion:**

Although uncommon, congenital cytomegalovirus infection should be included in the differential diagnosis of intraventricular hemorrhage and cardiomyopathy. Furthermore, this case highlights the possible severity of congenital cytomegalovirus infection, even in cases of previous maternal immunity.

## Background

Human cytomegalovirus (CMV) herpes-virus-5 is the most frequent cause of congenital infection, with a prevalence at birth of 0.64-2% [1]. Neonatal screening, confirming the diagnosis and investigating for the presence of possible sequelae, is vital in neonatology, as if treatment is appropriate, significant morbidity and mortality associated with complicated congenital infections can be reduced. CMV represents the top cause of non-hereditary mental retardation and non-genetic sensorineural deafness in childhood [2–4]. cCMV infection can cause serious complications in the fetus or in immunologically immature newborns, causing severe sequelae not only at birth but also later in childhood. Term infants who are infected typically present with no symptoms and have a lower chance of long-term sequelae because of their ability to control the infection. However, premature neonates or infants with primary immune disorders of T cells or natural killer cells, are at a higher risk, and may present with acute severe, potentially life-threatening symptoms that can result in permanent, irreversible sensorineural and cognitive disorders, and neurodevelopmental delay.

About 30–70% of women with primary CMV seroconversion and infection during pregnancy transmit the virus to their fetus [5]. The fetal damage depends mainly on the gestational age (GA) at which the fetal infection occurs. Women with prior immunity who are re-infected or have reactivation of CMV old infection have a much lower fetal transmission rate of 0.5-2% [1, 6].. Being immune therefore reduces the probability of maternal-to-fetal transmission of the virus. Non-primary infections can, however, cause serious damage to the developing fetus and have significant late sequelae in infancy and childhood [4, 7].

The diagnosis of congenital CMV infection requires detection of viral nucleic acid or replicating virus in the urine (or saliva) of the newborn during the first 3 weeks of life [6]. The detection of CMV DNA on the screening blood-spot test (Guthrie card) performed between 48 and 72 h after birth can be a valuable method to retrospectively confirm neonatal congenital CMV infection, if the test is positive. However, it should be noted that the sensitivity of this method is low [8, 9].

Neonates with signs of central nervous system infection (changes on cerebral ultrasound or abnormal hearing screening), chorioretinitis, or severe single- or multi-system disease should be treated to improve neurodevelopmental and audiological outcomes [10]. Antiviral therapy should be started within the first month of life and continued for six months [11]. Severely ill and symptomatic infants, and those not able to tolerate oral medications, are started on intravenous (IV) treatment with ganciclovir (6 mg/kg/dose IV twice daily), and then switched to oral valganciclovir (16 mg/kg/dose twice daily). The duration of therapy should be six months in total [12].

We describe a rare presentation of congenital cytomegalovirus from non-primary maternal infection, associated with intraventricular hemorrhage and cardiomyopathy, and we compare it to three similar case reports. Although uncommon, congenital cytomegalovirus infection should be included in the differential diagnosis of intraventricular hemorrhage and cardiomyopathy. Furthermore, this case highlights the possible severity of congenital cytomegalovirus infection, even in cases of previous maternal immunity.

## Case presentation

**Fig. 1:**
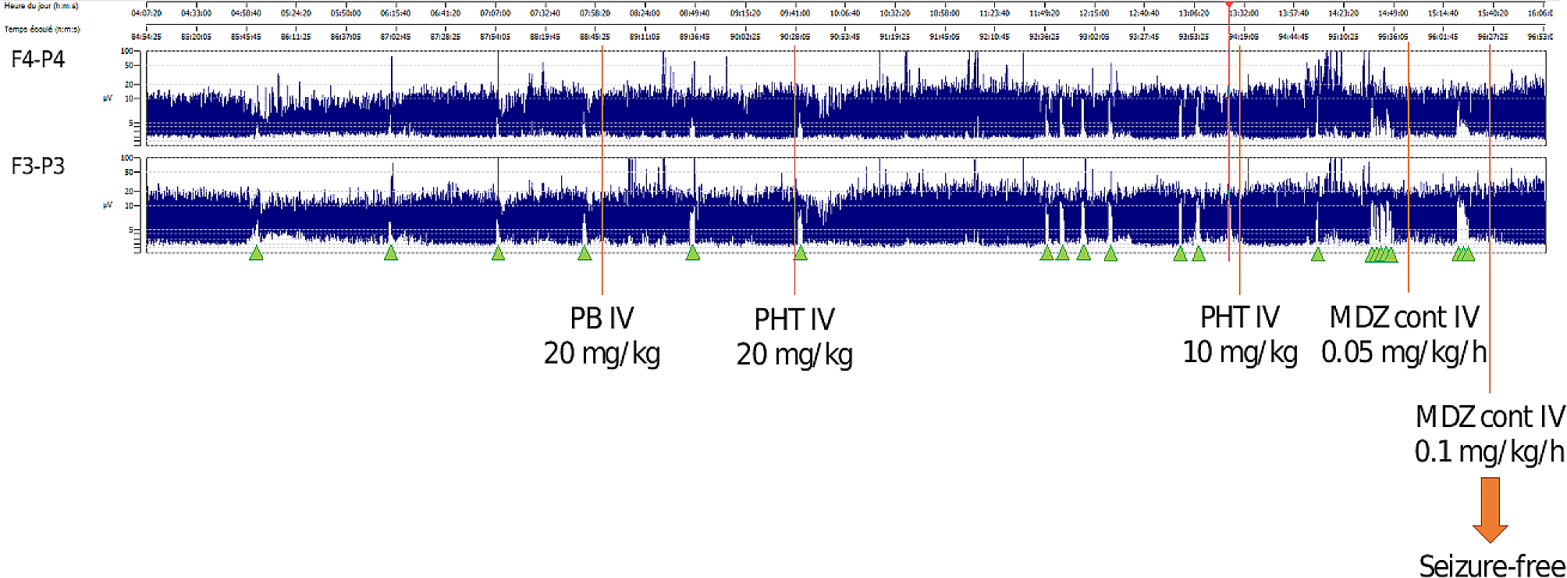
Amplitude integrated EEG in our patient PB - phenobarbital, PHT - phenytoin, MDZ– midazolam Green arrow: seizure

**Fig. 2:**
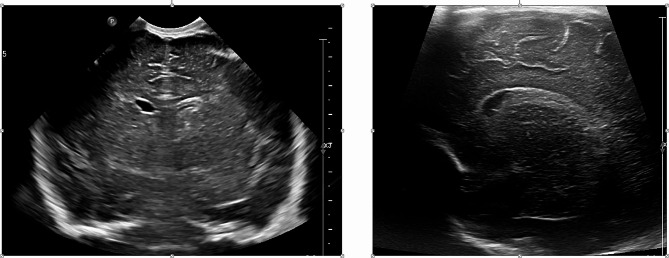
Brain ultrasonography

**Fig. 3:**
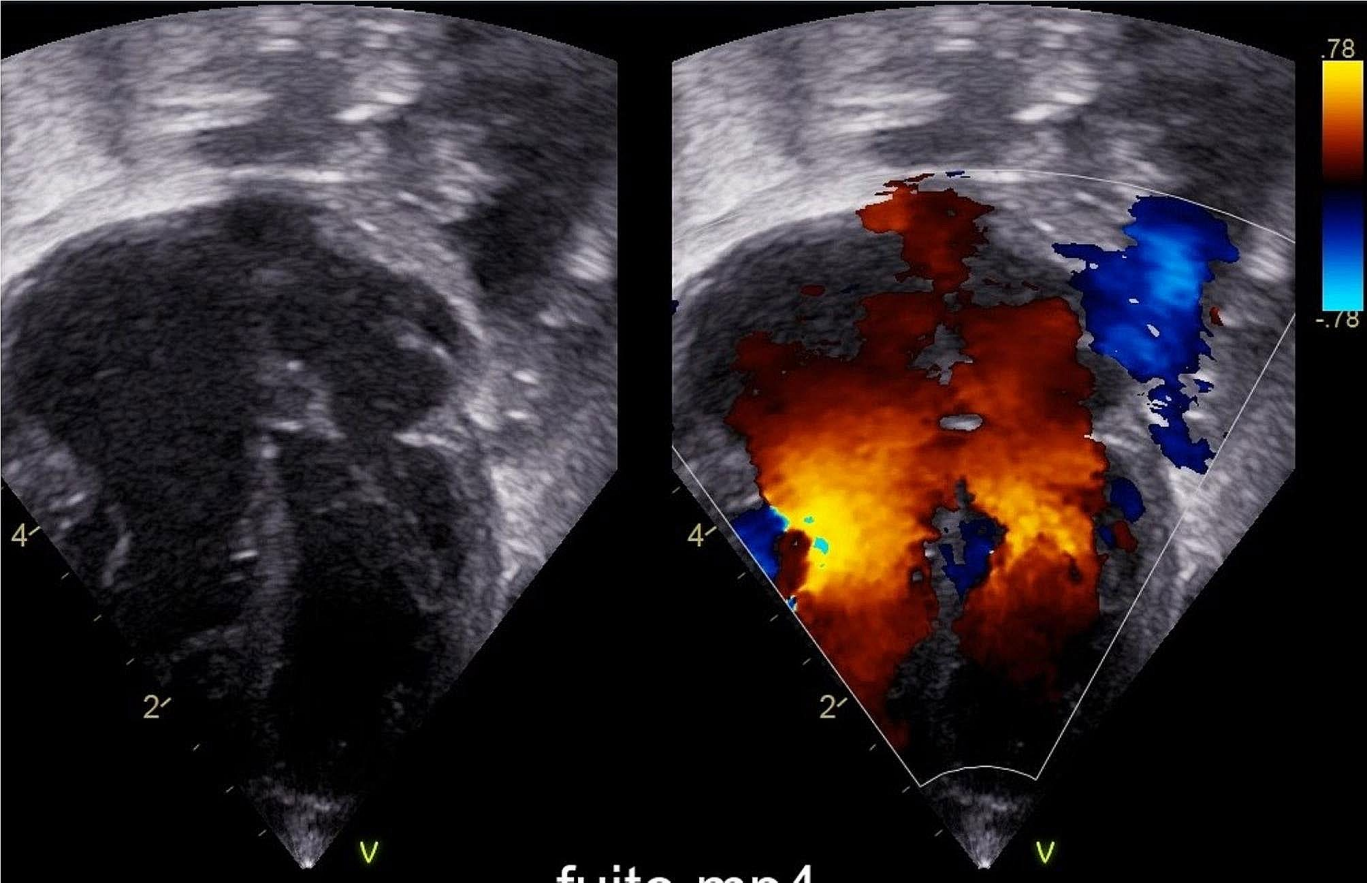
Echocardiography

**Fig. 4:**
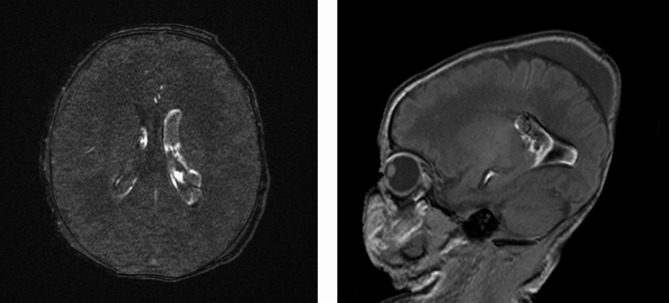
Brain Magnetic Resonance Imaging

An African full term male newborn was born at 37 weeks GA. Maternal medical history and her pregnancy course were unremarkable. Noninvasive prenatal testing (NIPT) was normal. Maternal serology in early pregnancy (8 weeks) showed immunization for rubella, cytomegalovirus (CMV IgG 112 U/mL, CMV IgM 7 U/mL), and toxoplasmosis. Screening for CMV reactivation was not performed during pregnancy. First and second trimester ultrasound scans (US) were normal. At 37 weeks and 5 days GA, the US showed polyhydramnios, fetal doppler reversal, and absence of fetal movements. As the cardiotocography (CTG) tracing revealed decreased heart rate variability, an emergency cesarean section was performed. At birth, the newborn was eutrophic (birth weight 2810 g (percentile 24), length 47.5 cm (percentile 24), and head circumference 34 cm (percentile 54)), and was bradycardic, apneic, and hypotonic. Apgar scores were at 1/4/9 at 1, 5 and, 10 min, respectively. The newborn was ventilated with positive pressure and supplemental oxygen for 5 min. Blood gas analysis at 1 h of life showed a pH of 7.12, lactate of 18.26 mmol/L, base excess − 15.5mmol/L and pC02 43.3mmHg. The clinical Thompson score was 7, suggestive of mild hypoxic ischemic encephalopathy (HIE) and the modified Sarnat was 2 (moderate HIE). The newborn fulfilled the criteria for TH and was transferred to our neonatal intensive care unit (NICU) at 3 h of life. On arrival, laboratory examination revealed the presence of thrombocytopenia (64,000/µl) and coagulopathy (INR 2.65), therefore platelet and plasma transfusions were given before the initiation of TH (4 h of life). Continuous full video-electroencephalogram (vEEG) monitoring showed normal electrical activity for the entire duration of TH. The blood result series are listed in Table 1. After 72 h of TH, rewarming was started (+ 0.5 °C per hour). Nine hours after the initiation of rewarming, he developed isolated apneic seizures. The vEEG showed left fronto-central rhythmic discharges corresponding to the episodes of apnea. A loading dose of intravenous phenobarbital (20 mg/kg) was administered but did not stop the seizures. A subsequent loading dose of phenytoin (20 mg/kg) was administered with no response. Due to the evolution into a status epilepticus, continuous intravenous midazolam was initiated and progressively increased to a maximum dose of 0.2 mcg/kg/min, followed by the resolution of seizures (Fig. 1). The vEEG monitoring was stopped 24 h after the last recorded seizure. A brain ultrasonography (US) was performed, showing a bilateral intraventricular hemorrhage with mild ventricular dilatation (grade III) (Fig. 2). The baby also presented hypotension and reduced capillary perfusion. Therefore, a sepsis screen was performed. Lab examination showed a mildly increased CRP of 9.7 mg/dl (negative value < 5 mg/dl), persistent thrombocytopenia (39,000/µl), and an ongoing mild coagulopathy (INR 1.36). Intravenous broad-spectrum antibiotic therapy was started. However, despite antibiotic and supportive therapy, the clinical evolution worsened, and on day 9 of life, an echocardiography revealed severe cardiac failure with an ejection fraction of 33% and signs suggestive of cardiomyopathy (Fig. 3). The cardiomyopathy screening, including metabolic and infectious screening of the blood, urine, cerebrospinal fluid (CSF), and nasal secretions, resulted in a positive Polymerase Chain Reaction (PCR) for CMV DNA in the CSF, nasal-pharyngeal swab, urine, and blood. The brain Magnetic Resonance Imaging (MRI) confirmed the bilateral ventricular hemorrhage, and the presence of ischemic-hemorrhagic spots in the frontal white matter and periventricular hyper echogenicity (Fig. 4). Thrombocytopenia was initially attributed to the HIE and to the TH-related thrombocytopenia and coagulopathy, but as it persisted despite platelet and plasma transfusions, it was probably related to CMV infection.

**Table 1:** Laboratory test results in the first week of life

	H1	H3	H24	H48	H72	H82	H96	D6
Hemoglobin	17 g/dl	19,4 g/dl	21.7 g/dl	20 g/dl	20.4 g/dl	18 g/dl	16,9 g/dl	16,5 g/dl
Platelet count	64 000/µl	64 000/µl	54 000/µl	44 000/µl	24,000/µl	39,000/µl	12,000/µl	105,000/µl
WBC	4700/ µl	15,940/µl	7400/µl	3800/µl	3400/µl	3640/µl	4100/µl	5600/µl
CRP	3 mg/dl	1,5 mg/dl	-	-	9,7 mg/L	8,7 mg/L	-	13,5 mg/L
INR	-	2,65	1,73	1,9	1,5	1,36	1,3	-
APTT	-	67,4	49,4	-	-	-	-	-
PT	-	30,6	19,8	-	-	-	-	-

H: age in Hours, D: age in Days

WBC: White Blood Cells, CRP: C reactive protein, INR: International Normalized Ratio, APTT: Activated Partial Thromboplastin Time, PT: Prothrombin Time

To determine the timing of the CMV infection, given that the mother did not undergo screening for CMV reactivation, we retrieved and tested the Guthrie card performed at birth for CMV DNA. The test was positive, demonstrating that the newborn had contracted intrauterine CMV infection due to maternal reactivation or re-infection. The child’s persistent thrombocytopenia, which was present also prior to cooling, and the cardiomyopathy were likely caused by congenital CMV infection. Due to the severity of the congenital infection, therapy with intravenous ganciclovir was initiated, and after 14 days, it was switched to oral valganciclovir for a total of 6 months. Auditory evoked potentials and fundoscopy at one month of age were normal. To date the baby is 12 months old. He had a normal developmental outcome at the 9 months follow-up but was unfortunately lost during the follow-up.

## Discussion and conclusions

In this case description, we present a rare manifestation of cCMV, characterized by cerebral hemorrhage and cardiomyopathy.

As the neonate presented with perinatal asphyxia, thrombocytopenia was initially attributed to asphyxia and therapeutic hypothermia (TH). The possibility of a CMV viral sepsis as the underlying cause of asphyxia and thrombocytopenia was not initially suspected in our patient, because of the previous maternal CMV immunization. In fact, the brain MRI findings in our patient did not suggest perinatal asphyxia. Severe HIE in term infants affects primarily gray matter, especially the basal ganglia and thalamus [13]. Instead, there was an involvement of the periventricular areas; the biventricular and frontal intraparenchymatous hemorrhages on MRI were not specific to cCMV but were rather due to thrombocytopenia and coagulopathy.

Only three other case reports were published in the last 10 years [14–16] about neonates with severe cCMV born by previously immune women. We compared them to our case (Table 2).

**Table 2:** Comparison of case reports about congenital cytomegalovirus infections due to non-primary maternal infection

	Stoykova 2020	Mack 2017	Arnouts 2022	Our case
Previously immune mother for CMV	Yes, for all			
Fetal ultrasonography	Not reported	subependymal cysts	normal	normal
Amniocentesis	none	CMV PCR 8.96 log GEq/ml	none	none
Term at birth	30 weeks	At term	At term	At term
gender	male	female	female	male
Apgar	1/1/3	8/8/10	6/6/7	1/4/9
Clinic at birth	petechial rash, generalized edema, hepatosplenomegaly	Extensive petechiae, hepatosplenomegaly, jaundice	mild respiratory distress, hepatosplenomegaly, petechiae, facial dysmorphic features, axial hypotonia.	bradycardia, apnea, hypotonia, mild hypoxic ischemic encephalopathy
Seizures	yes	no	no	yes
Thrombocytopenia	yes	yes	yes	yes
Suspected early onset sepsis	yes	no	yes	yes
Echocardiography	cardiomegaly and left ventricular hypertrophy	Not reported	Not reported	severe cardiac failure with an ejection fraction of 33% and signs suggestive of cardiomyopathy
Cerebral MRI	day 52 of life: extensive areas of encephalomalacia supratentorial bilateral with reduction of the brain parenchyma, pronounced dilatation of the ventricular system, evidence of massive intraventricular hemorrhage.	day 5 of life: bilateral subependymal cysts, mild dilatation of both lateral ventricles, white matter hyperintensities	widened symmetrical ventricles, subependymal germinolytic cysts, periventricular pseudocysts and micro-bleeding sequelae against the anterior horn of the lateral ventricles and at the occipital horn of the right lateral ventricle.	bilateral ventricular hemorrhage, presence of ischemic-hemorrhagic spots in the frontal white matter, periventricular hyper echogenicity
Cerebral ultrasonography	brain edema, calcification bilaterally, hyperechogenic foci in both sides of the ventricular wall, asymmetric ventriculomegaly	Not reported	bilateral germinolytic cysts with pronounced symmetric periventricular flaring, striatal vasculopathy, moderate dilatation of the ventricles	bilateral intraventricular hemorrhage with mild ventricular dilatation (grade III)
Treatment	ganciclovir IV 6 weeks	ganciclovir IV 3 weeks, then oral valganciclovir 3 weeks	ganciclovir IV 3 day then oral valganciclovir for 6 months	ganciclovir IV for 2 weeks, then oral valganciclovir for a total of 6 months.
Follow up	Not reported	4.5 years of age: normal neurological development but severe hearing loss on the right side.	4 months of age: central motor disorder with axial hypotonia and poor head control, hearing loss on the right side.	9 months of age: normal developmental outcome

Our case highlights the potential severity of cCMV infection even in previously immune mothers: the severe thrombocytopenia was responsible for a severe intraventricular hemorrhage and provoked a cardiomyopathy. Thrombocytopenia is a consistent finding in all the case reports and is well-known to be associated with cCMV infection. In the case report from Stoykova et al. [14], the newborn presented with a massive intraventricular hemorrhage shown by MRI, like in our case. Both cases that experienced cerebral hemorrhage also presented with seizures.

cCMV infection can mimic bacterial sepsis. In 3 out of 4 reported cases, including our own, treatment with broad-spectrum antibiotics was initiated [14, 16]. In the case described by Stoykova et al. [14], the patient was born at 30 weeks of gestational age with extremely severe general conditions and presented with an Apgar of 1/1/3 at 1, 5 and, 10 min, respectively. He had pale skin, petechial rash and generalized edema. Artificial ventilation was needed, and cardiac ultrasound showed evidence of cardiomegaly and left ventricular hypertrophy. In the case described by *Arnout et al.* [16] the patient was born at term. Apgar scores were 6,6 and 7, respectively. Physical examination showed mild respiratory distress, hepatosplenomegaly, petechiae, facial dysmorphic features and axial hypotonia. Non-invasive ventilation was needed, and empirical antibiotic therapy was started for suspected sepsis. In our case, the patient was born at term. Apgar scores were at 1/4/9. He fulfilled the criteria for TH and later developed seizures. All 3 cases could have been explained by bacterial sepsis. Because of the mothers’ CMV immune status, there was a delay in diagnosis in all 3 cases.

Cardiomyopathy and cardiac dysfunction due to CMV has often been described in adults and older children. There are very few cases described in the literature related to congenital infections.

Stoykova et al. [14], described a patient who presented cardiomegaly and left ventricular hypertrophy. Our patient had severe cardiac failure with an ejection fraction of 33% and signs suggestive of cardiomyopathy. It was only after discovering the cardiac involvement that we thought of performing a viral screening in our patient, which led to the late diagnosis of cCMV. CMV infection can lead to cardiomyopathy through several mechanisms. The virus may directly infect cardiac cells, causing inflammation and damage to the heart muscle. Additionally, CMV-induced immune responses can trigger an inflammatory cascade, contributing to the development of cardiomyopathy by disrupting the normal functioning of the heart tissue [17, 18].

Mothers that are immune to CMV before pregnancy are currently not screened for CMV reactivation, because of the low probability of maternal fetal transmission, ranging from 0.5 to 1.5% [5]. Nevertheless, in the unlikely case of transmission, the severity of congenital CMV remains high.

The possibility of cCMV infection as the underlying cause of thrombocytopenia, cerebral hemorrhage and septic status was not initially suspected in our patient. Incorporating universal neonatal screening for cCMV as a standard protocol could have expedited the diagnosis, potentially preventing the cerebral hemorrhage.

There is ongoing debate regarding universal CMV testing of all newborns at birth. Our case report highlights the importance of an early diagnosis of cCMV infection. In fact, the early diagnosis of cCMV can lead to the early initiation of antiviral therapies and reduce neurological impairments and sequelae.

According to the European Expert Consensus, newborns with a maternal history of CMV infection during pregnancy, those who are symptomatic, and infants with sensorineural hearing loss should be screened for CMV infection [12]. Studies have shown that approximately half of cCMV infections were due to maternal non-primary infection during pregnancy [4].

The diagnosis of cCMV infection requires the presence of viral nucleic acid or a replicating virus within the first three weeks of life. After this period, it can be difficult to assess whether the infection is postnatally acquired [6]. The dried blood spot (Guthrie card) is a reliable method for confirming cCMV infection retrospectively [8], but its low sensitivity limits its value as a screening test [19]. In our case, the patient’s mother’s CMV serologies were not repeated during pregnancy, but the presence of CMV DNA on the Guthrie card performed at birth, confirmed cCMV infection.

In conclusion, this case report highlights the insidious presentations of cCMV infection. Cerebral hemorrhage and cardiac damage are rarely described in the literature, but cCMV infection must be included in their differential diagnosis. This case also serves as a reminder that, although rare, congenital CMV infection in a newborn from an immunized mother can be extremely severe.

In our view, it is essential to reconsider the discussion surrounding the universal screening of newborns and the ongoing screening of immunized mothers.

## Data Availability

Not applicable.

## Abbreviations

cCMV: Congenital cytomegalovirus
CMV: Human cytomegalovirus
CSF: Cerebrospinal fluid
CTG: Cardiotocography
GA: Gestational age
HIE: Hypoxic ischemic encephalopathy
IV: Intravenous
MRI: Magnetic Resonance Imaging
NICU: Neonatal intensive care unit
NIPT: Noninvasive prenatal testing
PCR: Polymerase Chain Reaction
TH: Therapeutic hypothermia
US: Ultrasound scans
vEEG: Video-electroencephalogram
